# Supplementary material for: Genome-Wide Identification of Strawberry C2H2-ZFP C1-2i Subclass and the Potential Function of FaZAT10 in Abiotic Stress
Source: Int J Mol Sci. 2022 Oct 28;23(21):13079. doi: 10.3390/ijms232113079 (PMC9654774; doi:10.3390/ijms232113079)

Figure S1.Multiple sequence alignment of the C1-2i subclass in cultivated and wild strawberry

Frist zinc fingers domain

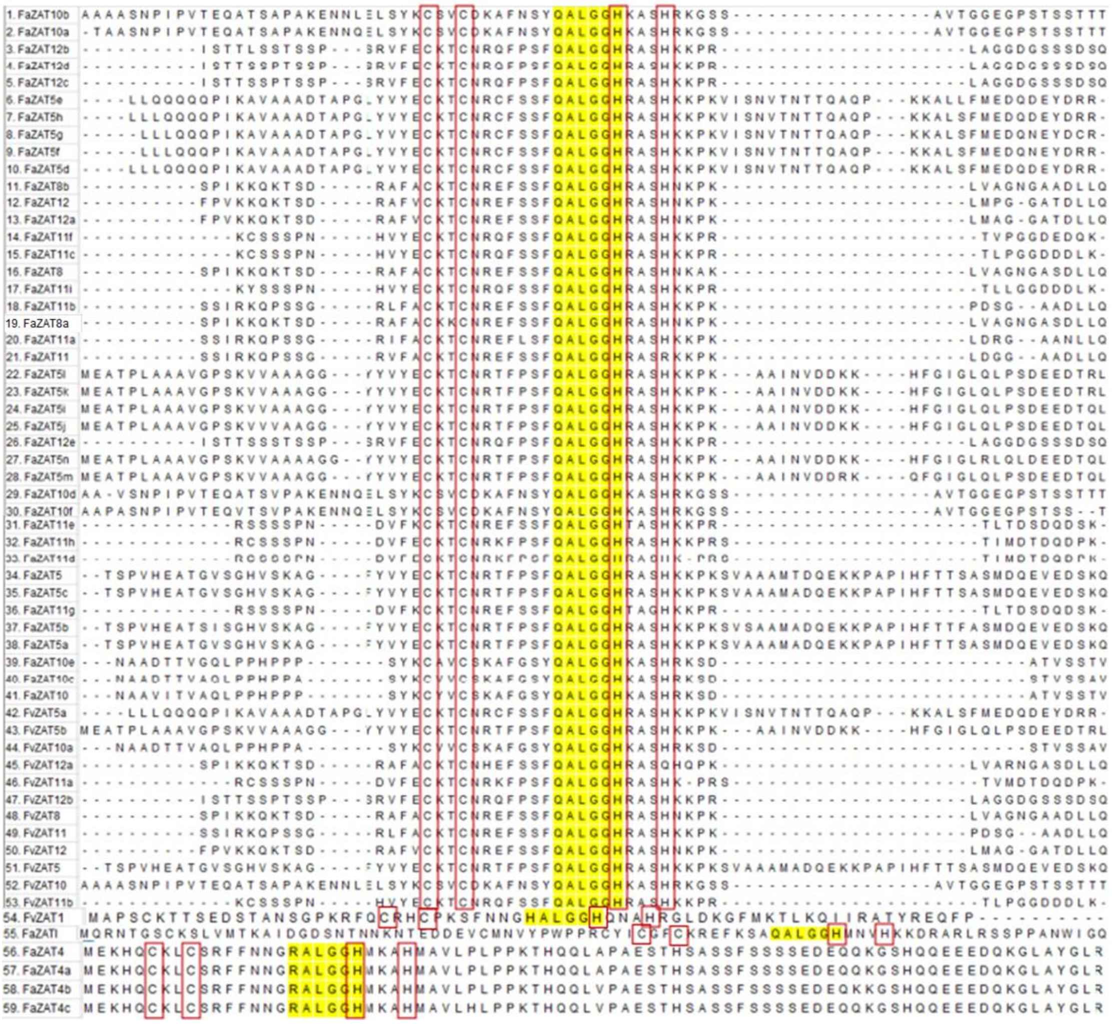

Second zinc fingers domain

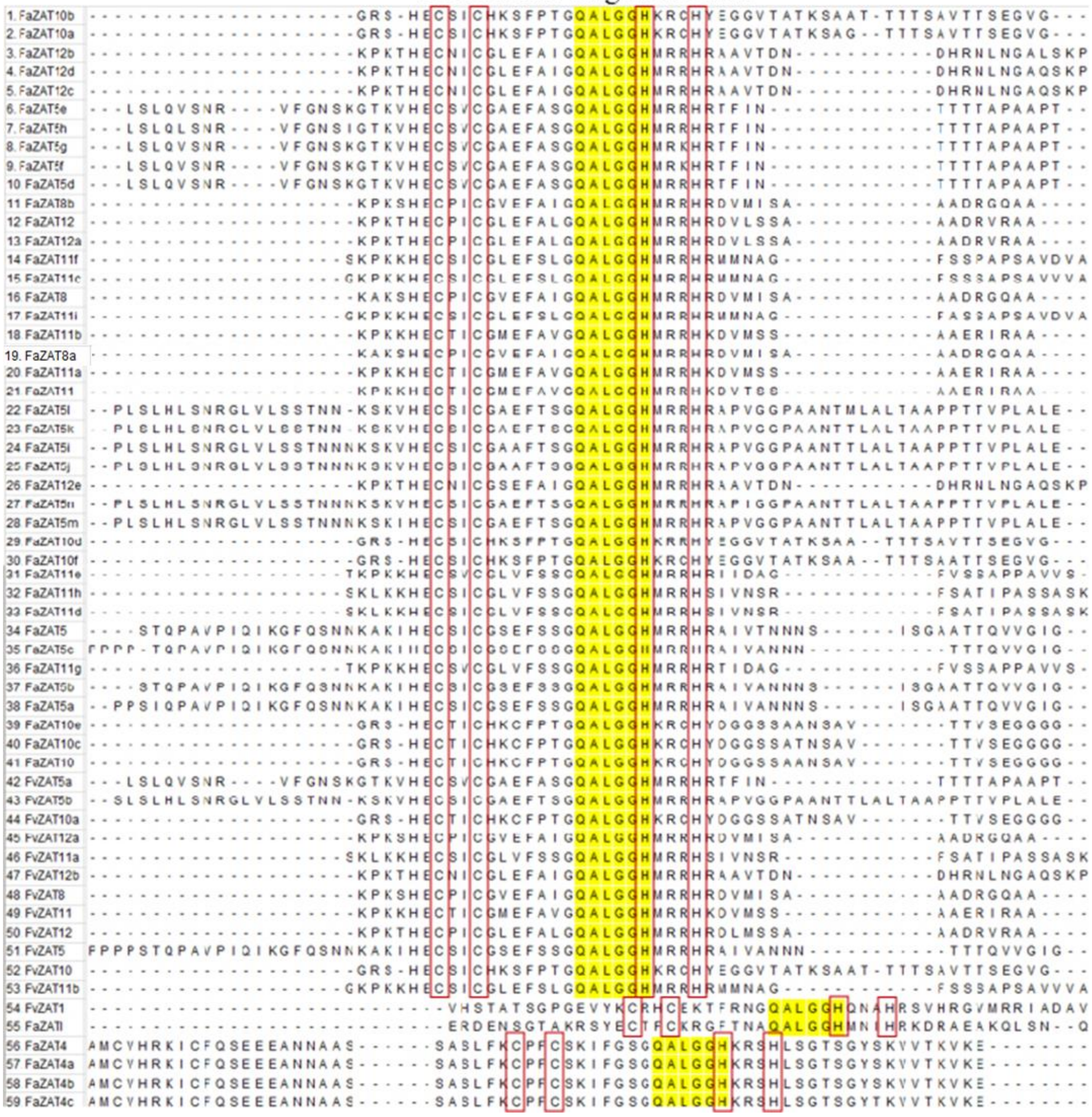

EAR domain

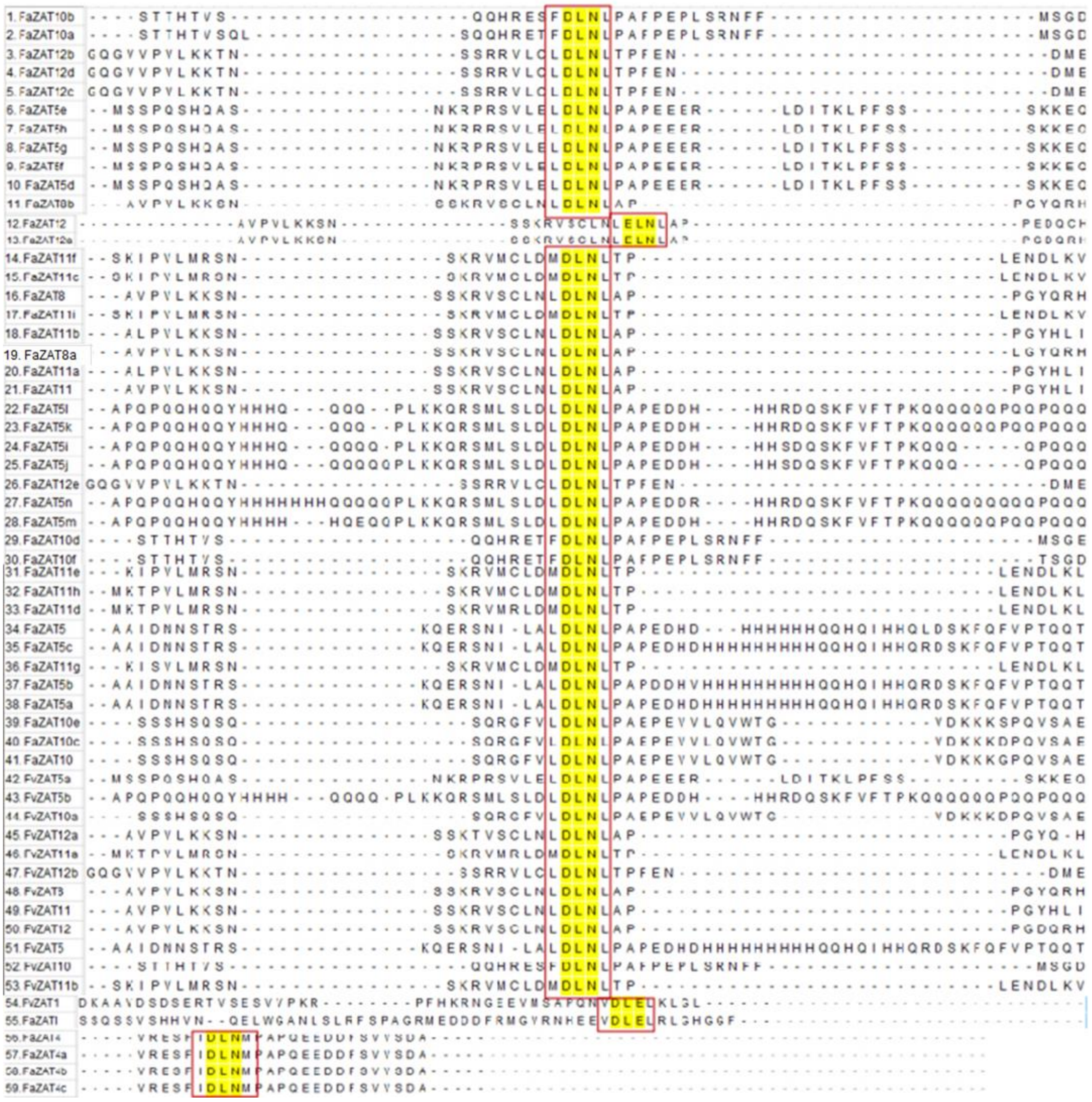

Supplement: Supplementary file 1 [file ijms-23-13079-s001.zip › Figure S1.pdf]
