# Supplementary material for: Genome-Wide Identification of Strawberry C2H2-ZFP C1-2i Subclass and the Potential Function of FaZAT10 in Abiotic Stress
Source: Int J Mol Sci. 2022 Oct 28;23(21):13079. doi: 10.3390/ijms232113079 (PMC9654774; doi:10.3390/ijms232113079)

Figure S2. The coding sequences and promoter sequences of *FaZAT10*

The sequence from start codon (ATG) to stop codon (TAG) of *FaZAT10*.

>*FaZAT10*

ATGGCTTTGGAAGCTCTCAACTCTCCCACCGCCGCCGGCCAAGGCTTTCACTTTGATG
AGCCCAAGCTCCAGTACTCGGAGCCATGGACCAAGCGCAAGCGCTCCAAGCGTCCAC
GAAGCGTCGAGCAGGCGGCCGCTCCGGTCTCCGAGGAGGAGTATCTCGCTCTCTGCCT
CATCATGCTCGCTCGCGGCGGTAACAGAGGACCACCCGTCACCACCACCACCACCGCC
GCCGCCGCCGCCGCCGCCTCAAACCCAATTCCGGTAACTGAACAGGCCACGTCAGCG
CAGGCCAAGGAGAATAACCTAGAGCTCAGTTACAAGTGCTCTGTTTGTGACAAGGCTT
TTAATTCCTACCAGGCTCTGGGTGGACACAAGGCCAGCCACAGGAAAGGGTCTTCCGC
CGTCACCGGCGGTGAAGGACCCTCCACGTCGTCGACTACCACCACCACATCCGCTGCG
ACTGTCTCAAACGCCTCAGGTAGGTCCCACGAGTGCTCCATCTGCCACAAGTCTTTCC
CCACCGGCCAGGCCTTGGGAGGGCACAAGCGTTGCCACTACGAGGGCGGCGTCACCG
CCACCAAGTCCGCCGCAACCACCACCACCAGCGCCGTGACCACCTCGGAGGGAGTGG
GGTCAACCACTCACACCGTCAGCCAACAACACCGTGAATCGTTTGATCTGAACTTACC
GGCCTTCCCGGAGCCTTTGTCGCGAAACTTCTTCATGTCCGGCGACGACGAGGTGGAA
AGCCCTCATCCGACGAAGAAGCCCCGTCTGTTCATGAGCCCCAAAGTTGAGATATCAC
TCAATTAG

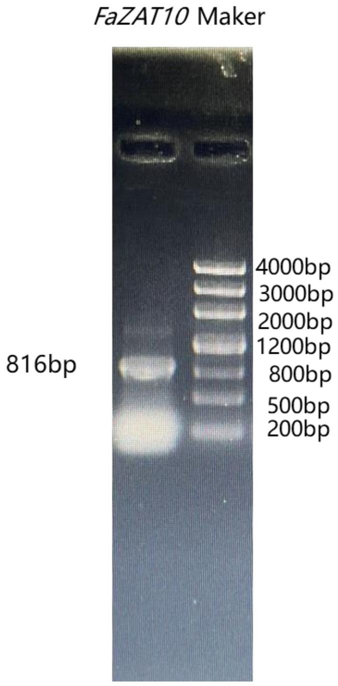

The promoter sequences of *FaZAT10*.

>*FaZAT10-Pro*

TCAGCTTAGAGTAAACAGTTTAGGCAACCCTTTTGAACGTTTTCACTTTCATCCAATTC
AAAACACAAGCACAGTAACTTCTAAATTACAGCATAACTAATCACGGACATTAATTTGC
TCCCAATTTACCACAACTATCAGACGTCAGGCTCAGCCGCTCAGGCTCATTCATATATGT
CGACCATGACTAAATAACAAATCTGATTACAGTAATTAAATTACGGGGGTGGTGAAAAT
TATAGAAATCTAGGCGATGATAGAAGCAACTAGTCAGCAAAACTTTTCCCTTTGATTAC

TTCAGTGATGATGATCTATATTATATTTAGTCGGCGGGGGGTACGTGTCTTTTTAGAAGA
TGCTGAAGCATGAGCCTCTCACTTTTCTAGCTAATTATATCGAAGCCAACCATAGAAGA
AAAGCCAGCCAACCCTCCTGAGTGAAGAGTCAAACAGATAAGAAGATCGAAGAAGAA
GAAGAAATCAACTTGGACAGTATTTAATACACGATCGATGACCAGTATACTACTACTGA
ACTCTCGGTCGACTCTCTCTGCTTCGAAGAACCGCTCGCTCTTTCTAGAAGTATCAAGT
GTGTGATAGCACATACGTACATCGTCAATATTATTATCCAGGTGTGACGCAATCGTGAAC
ACATATATATATAGTCATGAACGACCTTTCAATTTCGGGTGAGGCTGCGGAAACGTGTG
CATTGAATTAGAGCACACTATTGTGTATGATTTTATTCATCATAATAAGCAAGCAACCGC
TGAgGCAGCTCAGCAGAGACTCACGTCAATGTAGACGAGTCCATCGTATGCATCGATAT
TGTTCTCGACTTAATAATATTTGTCTACATAAGAAATTATCCGGTAATTGCGTACACATAA
ACAAAAGAAAAAGAAAAAAAAAAGGTAAGATATGTGGAAGTAGGAAAGGTCAACAGT
CCCTTCAAAATATCTAATTCGATGAGTGTGCTTAATTGGGATTTATTTTATATTAGGCTAA
TCAGATTTGAAAAGTTTAGTGATTAAAGGGAAACATAGGCAAATGGTATGCCTAATATT
AGGATGTACAAACTACCCCACAACCTCTCAACCACTGGTTAGAAGCACAGTATCCTCC
ACCGCCACTAACCCAAACCTCCCATGTGATCATCGACATGGCTAAGCAACCCCTTAATC
AATCCACACTTGAATTCTTAACACATTCATGCATGCATGCTTGACGGAACACCATACCC
AACTCCCCCAAATAAATCACAACTAACTATACAGCCTGTGAAGTGAGCCAAGGAAAAC
TCCCCGTGGTTACTGTGAATCCGTTAAAGCTTTTAATCGCAAATTACCGGAAGATTTATG
TGAATTTTCCGTAATGTACGGAGAGCCGTTAGATTAGTTACTATTTATGACGTTATTAAGT
AACCTTATATTCGTCGAAATTAGAAGCAATATAAAATATCTGAACACGGCTGCTACTGTT
TCGGATTTTGCTAATGAATCAGAGCCGGCTAAAGACCGAAGACCTGACCGCGTTTATTT
GGAAAAATTGCAATAAATTTGACTTTGAAGATGCCTTTGGGTTGGGCCCCGTAAACTTT
GTTTGGAGTTAAAAAGGGTGAAAACGGAGGCCCACCCTAAAACACTTGCACTCTTTG
ACCGACTTCGATTTTTGGTCATTCTTGGCCACCAAGAAAAGTAAAACGTGCTGCTCTC
ACTAACTTAGGTAAGTCTTTAATTTCGCAAAATCGGAAAAATAATAATAATTGTTAAATA
GGACACTTGGCCTTGGAGGACACTACTACTGCTTTCCTACCACTTGTCACGCAACTTCC
AACTCTCCCATCGCAACCCCTTCCCTTATAAACCCCCCCACTCTCTCCATTTCTCTTCAC
TCTCACTCATTCCAATCTCTTCTCTTCTCTCATTCACATAATACTCAACACTCCTCTCTC

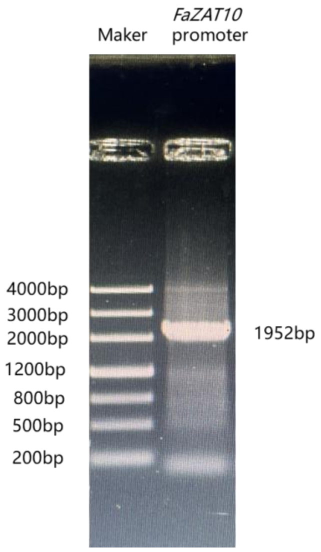

Supplement: Supplementary file 1 [file ijms-23-13079-s001.zip › Figure S2.pdf]
