# Supplementary material for: Genome-Wide Identification of Strawberry C2H2-ZFP C1-2i Subclass and the Potential Function of FaZAT10 in Abiotic Stress
Source: Int J Mol Sci. 2022 Oct 28;23(21):13079. doi: 10.3390/ijms232113079 (PMC9654774; doi:10.3390/ijms232113079)
Supplement: Supplementary file 1 [file ijms-23-13079-s001.zip › Figure S3.pdf]

Figure S3. Three highly conserved Motifs in cultivated strawberry C1-2i subclass

Motif 1:

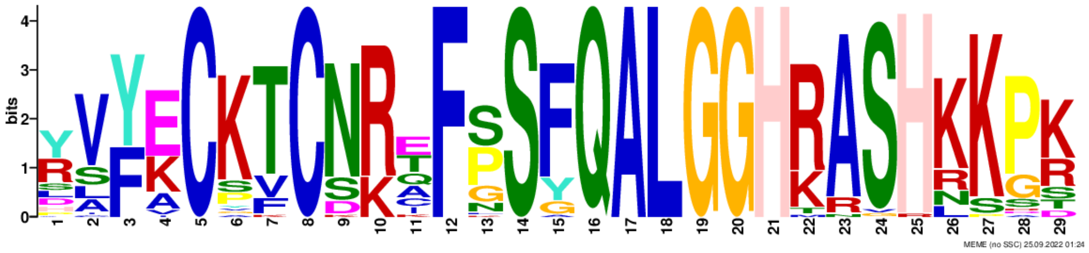

Motif 2:

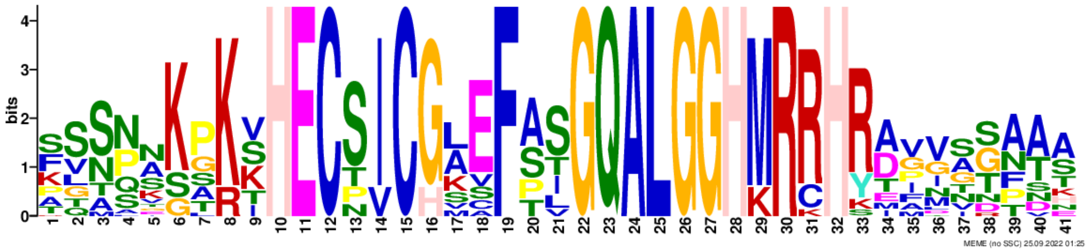

Motif 3:

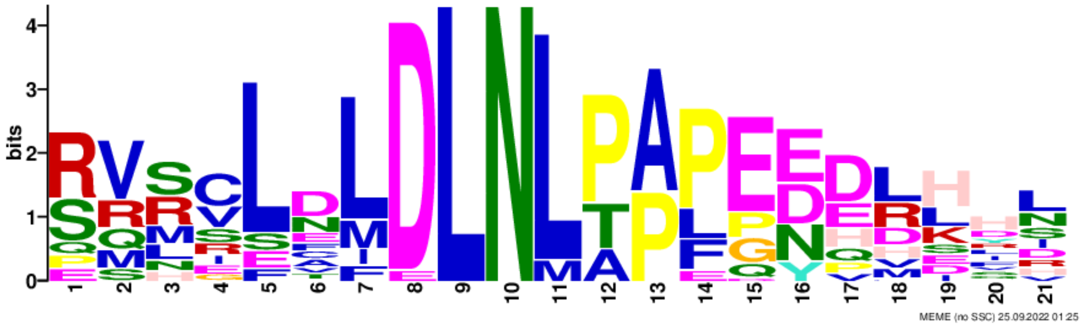

Motif sequence:

	Motif Consensus
Motif 1	YVYECKTCNREFSSFQALGGHRASHKKPK
Motif 2	SSSNNKPKVHECSICGLEFASGQALGGHMRRHRAVVSSAAA
Motif 3	RVSCLBLDLNLPAPEEDLHHL
